# Impact of COVID-19 on Online Interest in Urologic Conditions: An Analysis of Google Trends

**DOI:** 10.7759/cureus.21149

**Published:** 2022-01-12

**Authors:** Lakshay Khosla, Daniel Bockelman, Susan Gong, Gabriel Vizgan, Abdo E Kabarriti

**Affiliations:** 1 Department of Urology, State University of New York Downstate Health Sciences University, New York City, USA

**Keywords:** infodemiology, google trends, urology, social media, patient education as topic, online content, covid-19

## Abstract

Background

With COVID-19 leading to several isolation measures for preventative care, health care utilization, especially within urology, decreased substantially. The impact of COVID-19 on the population’s interests in urologic conditions remains to be established. By using the platform of Google Trends, which allows search behaviors and interest in healthcare topics to be quantified over time, we investigated the impact of COVID-19 on online search behaviors relating to common urologic conditions in the US.

Methods

The platform of Google Trends was utilized to analyze online interest in twelve common urologic conditions in the US from October 1, 2018 to August 1, 2021 (divided into “pre-COVID” and “COVID” periods at March 1, 2020). Search volume index (SVI), a measure of relative search volume on Google, data sets for the US, top queried and populated states, rising queries, and top queries were retrieved and analyzed for all conditions. Pre-COVID and COVID median SVIs were compared using the Mann Whitney U test, and correlations were analyzed using Spearman’s rank-order correlation test.

Results

For all twelve urologic conditions, rising and top queries were often related to symptoms, treatments, and COVID-19. COVID showed higher SVIs for erectile dysfunction (p=0.04) and lower SVIs for bladder cancer (p<0.01), hematuria (p<0.01), kidney cancer (p<0.01), kidney stones (p=0.03), and prostate cancer (p<0.01). Correlations to COVID-19 searches were seen for bladder cancer (R_S_=-0.36, p<0.01), erectile dysfunction (R_S_=0.20, p=0.04), hematuria (R_S_=-0.31, p<0.01), overactive bladder (R_S_=-0.23, p=0.04), and prostate cancer (R_S_=-0.33, p<0.01). No correlations were found for benign prostatic hyperplasia, interstitial cystitis, low testosterone, urinary incontinence, and urinary tract infections.

Conclusions

Online interest in many urologic conditions, especially cancers, decreased during COVID. Given the internet’s increasing role in healthcare, a reduced interest could translate to delayed diagnosis and treatment of these conditions. Only erectile dysfunction showed increasing interest, potentially due to research or misinformation linking it to COVID-19.

## Introduction

As COVID-19 spread globally, self-isolation, social distancing, and national lockdowns became crucial to control the pandemic. Due to stay-at-home orders, internet services have seen 40-100% rises in usage compared to pre-lockdown levels [1]. Specifically, there has been an increasing public interest in COVID-19 in the USA [2,3], especially displayed on the internet. Google Trends™ (Google LLC, Mountain View, California), a platform that quantifies search interests and trends on Google over time, is a powerful tool in analyzing public search patterns because Google accounts for greater than 70% of searches amongst other search platforms [4,5]. Since its launch in 2006, Google Trends has been used for healthcare-related research across various disciplines as the tool provides real-time insights into internet search behaviors by tracking and cataloging all Internet queries made through their web-based platforms. Data from these queries can then be used to study online health information-seeking behaviors, which patients engage in prior to appointments, and interest in various medical conditions and procedures, especially within urology [6,7].

Due to the highly personal nature of urologic conditions such as erectile dysfunction, prostate cancer, and urinary tract infection, the public will often “google” causes, symptoms, and treatments for their disorders [8,9]. In addition, the impact of COVID-19 on urologic conditions drew public concern and was propagated by social media [10-12], potentially due to the increasing use of the internet during the pandemic. Despite the variability in information accuracy and quality, internet use among men and women at risk of or diagnosed with urologic conditions makes it an important resource for decision-making, patient education, and support related to the disease [13].

Infodemiology is the science of distribution and determinants of information on the internet; data is leveraged to monitor online interest in health conditions and associated clinical implications [14]. Studies in urology have used infodemiology to determine that online interest in kidney stone surgery is constant despite the increasing prevalence and that interest in prostate cancer screening changed depending on The U.S. Preventive Services Task Force (USPSTF) guidelines [15,16]. However, the impact of COVID-19 on online search patterns for urologic conditions has not been investigated. The purpose of this study was to use Google Trends, a major search engine within the USA, to analyze internet search behaviors concerning the twelve most common Urologic conditions and their relation to COVID-19 at the national and state level.

## Materials and methods

Google Trends was queried for searches relating to the twelve most common urologic conditions and COVID-19 in the United States. The conditions analyzed included benign prostatic hyperplasia (BPH), bladder cancer, erectile dysfunction (ED), hematuria, interstitial cystitis, kidney cancer, kidney stones, low testosterone, overactive bladder (OAB), prostate cancer, urinary incontinence, and urinary tract infections (UTIs) [17,18]. Queries were limited to the USA and English language. The search period was from October 1, 2018 to August 1, 2021 to allow for analysis of searches 17 months before and 17 months after March 2020, when COVID-19 was declared a pandemic by the World Health Organization [19]. The midpoint was picked as March 1, 2020 because USA states started to declare a state of emergency and shut down at this point [20]. The period from October 1, 2018 to March 1, 2020 was marked “pre-COVID” (N=73 weeks), and from March 1, 2020 to August 1, 2021 was labeled “COVID” (N=75 weeks).

Google Trends gave data, split by week, on the search volume index (SVI), which is a measure of relative search volume on Google; the search is given a scaled value of 0 to 100, with 100 representing the peak search volume for the given search during the time period [4]. Searches analyzed on google trends were grouped phrases that allowed for all potential ways of searching a condition (e.g., “OAB” and “overactive bladder” would be incorporated under the search “bladder hyperactivity” when grouped as a “topic”). Data that was extracted included SVIs on the top five queried states (by proportion), populated states [21], rising queries (SVI not available), and top queries. Median SVIs were determined for the USA, top five queried, and top five populated states in the pre-COVID and COVID time periods.

The Mann-Whitney U test was used to compare pre-COVID to COVID median SVIs, and Spearman’s rank-order correlation was used to determine the correlation between searches related to each urologic condition and COVID-19 during the COVID time period. The p-value used for statistical significance in the analysis was <0.05. All analyses were done on SPSS v27.0 (IBM Inc., Armonk, New York).

## Results

Online interest in urologic conditions across the USA and various states was captured (Table 1). Rising queries and top queries for the conditions related to definitions, symptoms, management, treatments, and relation to COVID-19. Peak search (SVI=100) times in the USA were identified for the urologic conditions. Bladder cancer, hematuria, interstitial cystitis, kidney cancer, kidney stones, OAB, and prostate cancer searches had pre-COVID peaks. ED, low testosterone, urinary incontinence, and UTIs had COVID peaks. BPH had peaks for both pre-COVID and COVID time periods.

**Table 1:** Google Trends overview of urologic conditions from October 2018 to August 2021 SVI: search volume index; BPH: benign prostatic hyperplasia; COVID: coronavirus disease; TURBT: transurethral resection of bladder tumor; ED: erectile dysfunction; UTI: urinary tract infection; ICD: International Classification of Diseases; OAB: overactive bladder; PSA: prostate-specific antigen

Urologic condition	Top queried states (SVI)	Most populated states (SVI)	Rising queries	Top queries (SVI)
Benign prostatic hyperplasia	Florida (100)	California (76)	“Does an enlarged prostate affect a man sexually”	“Prostate” (100)
	Arizona (95)	Texas (73)	“Rezum treatment for BPH”	“BPH” (93)
	Connecticut (92)	Florida (100)	“Does ejaculation help enlarged prostate”	“Enlarged prostate” (77)
	Mississippi (91)	New York (90)	“Can an enlarged prostate cause ED”	“Prostatic” (23)
	Tennessee (91)	Pennsylvania (90)	“Urolift procedure”	“Benign” (21)
Bladder cancer	Maine (100)	California (44)	“Gemcitabine for bladder cancer”	“Symptoms” (100)
	New York (91)	Texas (44)	“Bladder cancer survival rates by age”	“Bladder cancer symptoms” (100)
	Vermont (86)	Florida (59)	“What are the signs of bladder cancer”	“Symptoms of bladder cancer” (46)
	Connecticut (84)	New York (91)	“Is bladder cancer treatable”	“Bladder cancer treatment” (44)
	New Hampshire (82)	Pennsylvania (69)	“TURBT bladder cancer”	“Bladder cancer signs” (38)
Erectile dysfunction	Louisiana (100)	California (57)	“Erectile dysfunction covid”	“Is erectile dysfunction” (100)
	West Virginia (93)	Texas (68)	“Covid 19 erectile dysfunction”	“ED” (61)
	Mississippi (90)	Florida (71)	“Coronavirus erectile dysfunction”	“Erectile dysfunction causes” (60)
	Kentucky (87)	New York (66)	“Curved erectile dysfunction”	“Erectile dysfunction help” (54)
	South Carolina (87)	Pennsylvania (71)	“Covid and erectile dysfunction”	“What is erectile dysfunction” (54)
Hematuria	West Virginia (100)	California (65)	“Covid blood in urine”	“Blood urine” (100)
	Mississippi (97)	Texas (72)	“Microscopic hematuria in females”	“Blood in urine” (92)
	New Mexico (94)	Florida (85)	“Blood in urine prostate cancer survivor”	“Hematuria” (42)
	North Dakota (93)	New York (73)	“What does blood in the urine indicate”	“Blood in urine cause” (9)
	Louisiana (93)	Pennsylvania (86)	“Nitrofurantoin”	“UTI” (9)
Interstitial cystitis	South Dakota (100)	California (55)	“Interstitial cystitis association”	“Cystitis” (100)
	Tennessee (82)	Texas (54)	“Chronic cystitis ICD-10”	“Interstitial cystitis” (97)
	Kentucky (81)	Florida (69)	“Pelvic floor dysfunction”	“Interstitial” (93)
	Wyoming (77)	New York (48)	“Bladder pain relief”	“Bladder” (50)
	Oklahoma (74)	Pennsylvania (82)	“Appendicitis”	“Bladder pain” (40)
Kidney cancer	West Virginia (100)	California (45)	“Stage 5 kidney cancer”	“Kidney” (100)
	Vermont (94)	Texas (59)	“History of kidney cancer ICD-10”	“Kidney cancer” (93)
	New York (85)	Florida (63)	“Kidney cancer treatment options”	“Kidney cancer symptoms” (22)
	Wyoming (82)	New York (85)	“Is kidney cancer genetic”	“Symptoms” (22)
	Pennsylvania (73)	Pennsylvania (73)	“History of renal cancer ICD-10”	“Renal cancer” (18)
Kidney stones	West Virginia (100)	California (52)	“Stages of passing a kidney stone”	“Kidney” (100)
	Kentucky (90)	Texas (63)	“How to pass a kidney stone at home”	“Kidney stones” (68)
	Tennessee (89)	Florida (68)	“How to pass a kidney stone in 24 hours”	“Kidney stone” (41)
	Alabama (84)	New York (58)	“Best way to pass a kidney stone”	“Kidney pain” (12)
	Oklahoma (80)	Pennsylvania (76)	“What causes kidney stones”	“Symptoms” (10)
Low testosterone	Wyoming (100)	California (42)	“Does low testosterone cause ED”	“Low testosterone men” (100)
	Alabama (94)	Texas (79)	“Does low testosterone cause infertility”	“Symptoms” (92)
	Oklahoma (94)	Florida (65)	“Can women have low testosterone”	“Testosterone low symptoms” (89)
	Mississippi (88)	New York (48)	“How can you tell if you have low testosterone”	“Low testosterone in men” (72)
	Tennessee (87)	Pennsylvania (50)	“How do you know if your testosterone is low”	“Can low testosterone” (64)
Overactive bladder	South Dakota (100)	California (46)	“Frequent urination causes”	“Overactive bladder” (100)
	Arkansas (86)	Texas (53)	“What is an overactive bladder”	“OAB” (33)
	Mississippi (86)	Florida (60)	“OAB Treatment”	“ICD-10 overactive bladder” (8)
	Delaware (84)	New York (72)	“Signs of overactive bladder”	“Overactive bladder symptoms” (7)
	Nebraska (83)	Pennsylvania (68)	“Overactive kidneys”	“Overactive bladder medication” 97)
Prostate cancer	New York (100)	California (69)	“What percent of men get prostate cancer”	“Prostate” (100)
	Kansas (90)	Texas (68)	“Encounter for chemotherapy for prostate cancer”	“Cancer” (93)
	Florida (86)	Florida (86)	“Cyberknife for prostate cancer locations”	“Prostate cancer” (87)
	New Jersey (85)	New York (100)	“Does masturbation cause prostate cancer”	“PSA” (11)
	Pennsylvania (84)	Pennsylvania (84)	“When should men get a prostate exam”	“Symptoms” (9)
Urinary incontinence	New Hampshire (100)	California (71)	“Best incontinence underwear for women”	“Incontinence” (100)
	West Virginia (98)	Texas (72)	“Attain for incontinence”	“Bladder” (24)
	Vermont (92)	Florida (80)	“Attain incontinence device”	“Urinary incontinence” (20)
	Alabama (91)	New York (90)	“Incontinence supplies near me”	“Bladder control” (14)
	Mississippi (90)	Pennsylvania (84)	“Bladder control underwear”	“Incontinence pads” (7)
Urinary tract infections	Mississippi (100)	California (71)	“Does doxycycline treat UTI”	“UTI” (100)
	West Virginia (100)	Texas (78)	“What causes UTIs in women”	“Symptoms” (18)
	Alabama (96)	Florida (79)	“Can boys get UTIs”	“Symptoms UTI” (15)
	Arkansas (90)	New York (65)	“Can UTI affect your period”	“Urinary tract” (12)
	Tennessee (89)	Pennsylvania (78)	“Best antibiotic for UTI”	“Bladder infection” (11)

**Figure 1:**
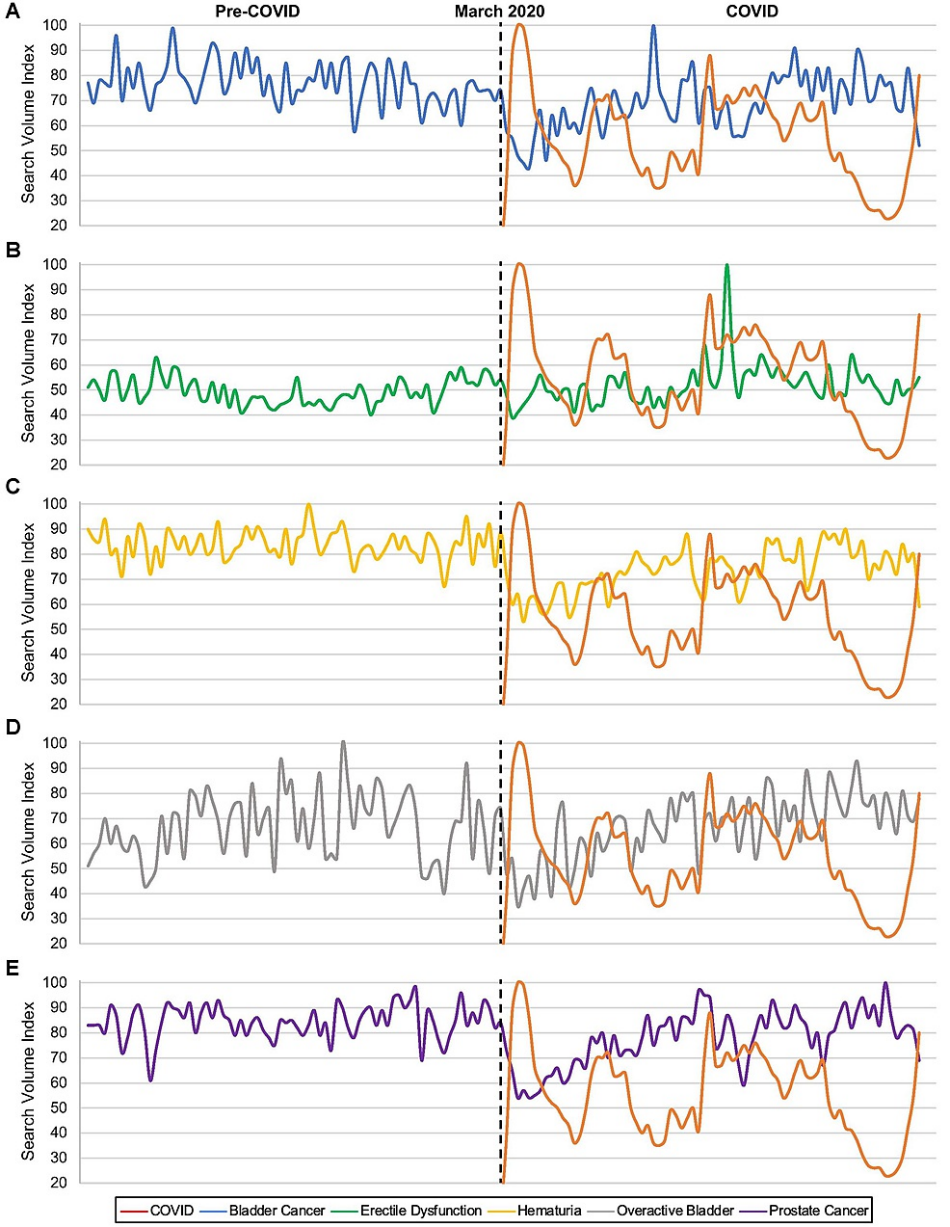
Online interest in urologic conditions showing significant correlation with COVID-19 related searches Urologic conditions showing significant correlation with COVID-19 related searches include (A) bladder cancer, (B) erectile dysfunction, (C) hematuria, (D) overactive bladder, and (E) prostate cancer.

During the COVID period in the USA, median SVI was significantly higher for erectile dysfunction (p=0.04) and lower for bladder cancer (p<0.01), hematuria (p<0.01), kidney cancer (p<0.01), kidney stones (p=0.03), and prostate cancer (p<0.01) (Table 2). Significant negative correlations to COVID searches were identified for bladder cancer (R_S_=-0.36, p<0.01), hematuria (R_S_=-0.31, p<0.01), OAB (R_S_=-0.23, p=0.04), and prostate cancer (R_S_=-0.33, p<0.01) and a significant positive correlation to COVID searches was present for ED (R_S_=0.20, p=0.04) (Table 3 and Figure 1). Data comparing median SVIs of pre-COVD to COVID and Spearman's correlations of COVID search terms to disease-specific terms for the top five populated and top five queried states is found in the Appendix. 

**Table 2:** Comparison of Median Search Volume Index of Urologic Conditions Pre-COVID and during COVID in the United States. * Indicates p<0.05

Urologic condition	Pre-COVID; median (IQR)	COVID; median (IQR)	P
Benign prostatic hyperplasia	82.0 (11.0)	82.0 (14.0)	0.87
Bladder cancer *	76.0 (11.0)	69.0 (13.5)	<0.01
Erectile dysfunction *	48.0 (7.0)	51.0 (8.5)	0.04
Hematuria *	83.0 (8.0)	74.0 (11.5)	<0.01
Interstitial cystitis	73.0 (13.0)	70.0 (14.5)	0.12
Kidney cancer *	48.0 (10.0)	41.0 (13.5)	<0.01
Kidney stones *	85.0 (7.0)	83.0 (8.0)	0.03
Low testosterone	70.0 (13.0)	69.0 (18.0)	0.66
Overactive bladder	69.0 (20.0)	69.0 (17.0)	0.93
Prostate cancer *	85.0 (8.0)	80.0 (15.5)	<0.01
Urinary incontinence	73.0 (6.0)	76.0 (16.5)	0.23
Urinary tract infections	86.0 (5.0)	85.0 (6.5)	0.06

**Table 3:** Correlation of urologic condition searches to COVID-19 related searches during COVID in the United States * Indicates p<0.05

Urologic Condition	R_S_	P
Benign prostatic hyperplasia	-0.18	0.12
Bladder cancer *	-0.36	<0.01
Erectile dysfunction *	0.20	0.04
Hematuria *	-0.31	<0.01
Interstitial cystitis	-0.21	0.07
Kidney cancer	-0.10	0.40
Kidney stones	-0.19	0.11
Low testosterone	-0.21	0.08
Overactive bladder *	-0.23	0.04
Prostate cancer *	-0.33	<0.01
Urinary incontinence	-0.22	0.05
Urinary tract infections	0.02	0.91

## Discussion

Online search engine trends, specifically Google Trends, have proven useful indicators of shifts in the public’s interest in healthcare-related topics and how they correlate with major events [15,22]. For example, in 2019, Rezaee et al. [16] determined differences in online interest in prostate cancer diagnosis, screening, and treatments before and after different USPSTF guidelines were released for prostate cancer. With the COVID-19 pandemic becoming mainstream in the United States in March 2020 [20], the landscape of all medical fields, including urology, was significantly impacted. To our knowledge, this study is the first to provide a robust analysis of Google Trends data to analyze the impact of COVID-19 on the most common urologic conditions.

By analyzing Google Trends data, we saw that the most populated states were not the states with the highest search interest proportional to population, and many of the top five by proportion states overlapped between conditions. For example, states like Mississippi (seven), West Virginia (five), and Tennessee (five) were listed as the top five states for at least five of the conditions we queried. There are many potential factors that could influence increased public interest in urologic conditions. There may be a higher disease prevalence in these states, thus driving more online interest in these conditions. Similarly, interest in associated urologic and non-urologic conditions within the state can play a role; states with a higher prevalence of cardiovascular disease may have higher searches related to ED [23]. It could also be related to the locations of academic centers or hospitals that focus heavily on these urologic conditions and therefore drive more interest in those geographical areas. Major academic hospitals tend to be clustered in the most populated states [24]; this is reflected by more overlap of the most populated states with the top states for search interest in urologic cancers, which often may require patients to visit these major academic centers. Nevertheless, further exploration into what drove online interest in urologic conditions in these areas is warranted.

Google Trends also provided us with data on the top queries and rising queries related to the conditions, providing insight into public interest in these conditions. Specifically, treatments were a part of rising or top queries for nine of the twelve conditions, including all three malignant conditions. Symptoms were also a large focus for most of the searches. Importantly for this study, there were two conditions that had rising queries related to coronavirus: ED and hematuria. ED specifically had a strong correlation with rising queries and COVID-19, with four out of the top five rising queries being related to COVID; this was likely due to research suggesting links between the two conditions or a spread of misinformation on the internet [12]. Similarly, hematuria and COVID are linked through potential mechanisms of acute kidney injury and could explain the rising COVID-related queries for the condition [25].

Online interest in most urologic conditions (especially bladder cancer, hematuria, kidney cancer, kidney stones, OAB, and prostate cancer) marked by lower median SVIs during COVID or a negative correlation to COVID related searches was noted to decrease in the COVID time period compared to the pre-COVID period at the national and state level. These decreases likely are the result of patients not being adequately diagnosed during this time period, as people were avoiding doctors’ offices and public settings during the height of the pandemic in the hopes of avoiding infection [26,27]. Due to a lack of diagnosis regardless of the maintained prevalence, public interest in many urologic conditions was seen to decrease, marked by the decreasing online interest. Unfortunately, this time period could have led to a proportion of these would-be patients to have progressed in their respective diseases, making this clinically noteworthy. The only condition to show significantly increased online interest during the pandemic was erectile dysfunction. This suggests a link between ED and COVID in the public’s viewpoint, potentially stemming from research indicating links between the conditions [12,28], a spread of misinformation linking COVID or vaccines to ED [29], or a mix of the two.

Graphical representations of urologic condition searches showing significant correlations to COVID-19 searches demonstrate a trend in which searches for COVID-19 peak while the searches for the urological conditions downtrend (with the exception of ED) (Figure 1). This fluctuation of COVID-19 searches could be related to “waves” of COVID-19 throughout the United States or significant developments in treatment or vaccination news [20]. This decreased interest in most urologic conditions, especially cancers like bladder and prostate cancer, during these waves likely exacerbated delays in diagnosing and treating these conditions, the impact of which may be experienced by the urology community as things return to normal [26]. Further analysis into the specific timing of these peak searches could provide more insight into factors that changed public interest.

There are important limitations to this study. Firstly, this research paper did not analyze searches on any other online search databases (e.g., Yahoo, Bing) or analyze searches on any social media websites (e.g., Twitter, Facebook), potentially leading to a lack of trends that were present in alternate search engines. Furthermore, it is difficult to distinguish search interests by subgroups (e.g., potential patients, doctors, students), and therefore associations noted may be diminished or supplemented. This is a descriptive study, and the findings do not imply causation, especially because there is a lack of clinical data. Clinical data, if available, could help supplement findings; nevertheless, we included analysis on many months of data across the USA to strengthen findings. A lack of analysis into international trends may also make it difficult to apply the findings to other countries. However, a lack of access to the internet in various countries would add in additional confounders that would diminish trends noted.

## Conclusions

In conclusion, online interest in bladder cancer, hematuria, kidney cancer, kidney stones, OAB, and prostate cancer decreased during COVID compared to the pre-COVID time period. The decreased interest in these conditions meant that focus was shifted away from these conditions, potentially leading to delays in diagnosis and treatment; the impact of the delays may be felt by the urology community in the coming months as things return to normal. Erectile dysfunction was the only condition to show an increased online interest, potentially due to research linking it to COVID-19 or the spread of misinformation linking it to COVID-associated factors like vaccines.

## Appendices

Tables 4 provides data comparing median search volume index (SVI) between Pre-COVID and during COVID in the top five queried and top five most populated states for urologic conditions. Table 5 provides data on the correlation of urologic condition searches to COVID-19 related searches during COVID in the top five queried and top five most populated states. 

**Table 4:** Comparison of median search volume index of urologic conditions pre-COVID and during COVID in top queried and most populated states * Indicates p<0.05

Urologic condition	Location	Pre-COVID; median (IQR)	COVID; median (IQR)		P
Benign prostatic hyperplasia	Top queried states:				
	Florida	54.0 (21.0)		52.0 (19.0)	0.85
	Arizona	31.0 (21.0)		23.0 (22.5)	0.06
	Connecticut	29.0 (18.0)		27.0 (24.0)	0.31
	Mississippi	30.0 (37.0)		29.0 (33.0)	0.65
	Tennessee	35.0 (22.0)		30.0 (22.5)	0.10
	Most populated states:				
	California	59.0 (17.0)		59.0 (23.5)	0.69
	Texas *	55.0 (24.0)		61.0 (25.0)	0.04
	Florida	54.0 (21.0)		52.0 (19.0)	0.85
	New York	61.0 (33.0)		62.0 (21.0)	0.85
	Pennsylvania	40.0 (30.0)		41.0 (23.5)	0.73
Bladder cancer	Top queried states:				
	Maine	23.0 (33.0)		22.5 (32.0)	0.66
	New York	54.0 (21.0)		55.0 (19.5)	0.73
	Vermont	12.0 (32.0)		9.0 (35.0)	0.07
	Connecticut	14.0 (15.0)		13.0 (26.0)	0.29
	New Hampshire	10.0 (19.0)		10.0 (18.0)	0.95
	Most populated states:				
	California	50.0 (19.0)		45.0 (22.5)	0.08
	Texas *	49.0 (21.0)		44.0 (18.0)	0.01
	Florida	53.0 (28.0)		51.0 (24.5)	0.21
	New York	54.0 (21.0)		55.0 (19.5)	0.73
	Pennsylvania	46.0 (30.0)		37.0 (19.5)	0.06
Erectile dysfunction	Top queried states:				
	Louisiana	25.0 (29.0)		22.0 (24.0)	0.08
	West Virginia *	25.0 (21.0)		29.0 (14.0)	<0.01
	Mississippi *	19.0 (23.0)		26.0 (16.0)	<0.01
	Kentucky	30.0 (30.0)		32.0 (34.5)	0.20
	South Carolina	32.0 (32.0)		30.0 (29.0)	0.29
	Most populated states:				
	California	50.0 (12.0)		49.0 (10.5)	0.42
	Texas *	41.0 (11.0)		46.0 (11.0)	<0.01
	Florida *	50.0 (12.0)		55.0 (14.5)	0.03
	New York	58.0 (21.0)		55.0 (17.5)	0.71
	Pennsylvania	33.0 (19.0)		31.0 (19.0)	0.38
Hematuria	Top queried states:				
	West Virginia *	22.0 (20.0)		18.0 (29.0)	0.04
	Mississippi	15.0 (35.0)		13.0 (14.5)	0.13
	New Mexico	17.0 (32.0)		15.0 (28.0)	0.06
	North Dakota	15.5 (37.0)		14.0 (32.0)	0.01
	Louisiana	30.0 (27.0)		22.0 (25.5)	0.12
	Most populated states:				
	California	47.0 (29.0)		47.0 (28.0)	0.46
	Texas *	56.0 (16.0)		46.0 (18.5)	<0.01
	Florida *	62.0 (22.0)		55.0 (15.5)	<0.01
	New York *	64.0 (20.0)		56.0 (24.0)	<0.01
	Pennsylvania	42.0 (19.0)		41.0 (23.0)	0.79
Interstitial cystitis	Top queried states:				
	South Dakota	9.0 (17.0)		11.0 (19.0)	0.58
	Tennessee	41.0 (40.0)		36.0 (37.0)	0.05
	Kentucky	23.0 (39.0)		22.0 (40.5)	0.49
	Wyoming	4.0 (15.5)		9.0 (22.5)	0.10
	Oklahoma	24.0 (27.0)		21.0 (25.5)	0.31
	Most populated states:				
	California	37.0 (19.0)		38.0 (22.5)	0.75
	Texas	35.0 (27.0)		33.0 (22.5)	0.39
	Florida	44.0 (26.0)		38.0 (27.0)	0.73
	New York *	36.0 (23.0)		32.0 (24.5)	0.04
	Pennsylvania	38.0 (26.0)		33.0 (25.0)	0.10
Kidney cancer	Top queried states:				
	West Virginia	14.0 (31.0)		9.0 (24.5)	0.07
	Vermont	13.0 (31.0)		12.0 (22.5)	0.83
	New York *	40.0 (20.0)		35.0 (17.5)	<0.01
	Wyoming	6.5 (19.0)		5.0 (14.5)	0.81
	Pennsylvania	35.0 (23.0)		33.0 (26.0)	0.65
	Most populated states:				
	California *	54.0 (35.0)		41.0 (23.5)	<0.01
	Texas *	37.0 (20.0)		31.0 (20.0)	0.04
	Florida	37.0 (20.0)		35.0 (22.5)	0.60
	New York *	40.0 (20.0)		35.0 (17.5)	<0.01
	Pennsylvania	35.0 (23.0)		33.0 (26.0)	0.65
Kidney stones	Top queried states:				
	West Virginia	43.0 (29.0)		39.0 (24.5)	0.52
	Kentucky *	50.0 (18.0)		43.0 (20.5)	<0.01
	Tennessee *	58.0 (22.0)		53.0 (21.5)	0.02
	Alabama	58.0 (25.0)		56.0 (22.5)	0.23
	Oklahoma *	46.0 (22.0)		38.0 (22.5)	0.04
	Most populated states:				
	California	72.0 (13.0)		73.0 (12.0)	0.65
	Texas *	73.0 (13.0)		68.0 (14.0)	<0.01
	Florida *	69.0 (12.0)		65.0 (14.5)	0.02
	New York	72.0 (18.0)		69.0 (17.5)	0.10
	Pennsylvania *	65.0 (14.0)		58.0 (17.0)	0.03
Low testosterone	Top queried states:				
	Wyoming *	12.0 (23.5)		4.0 (16.0)	0.01
	Alabama	30.0 (21.0)		28.0 (32.0)	0.88
	Oklahoma	14.0 (27.0)		12.0 (24.0)	0.06
	Mississippi	14.0 (21.0)		13.0 (15.0)	0.09
	Tennessee	28.0 (18.0)		32.0 (19.0)	0.62
	Most populated states:				
	California	34.0 (14.0)		38.0 (20.0)	0.64
	Texas	54.0 (20.0)		54.0 (27.0)	0.65
	Florida	41.0 (24.0)		38.0 (22.5)	0.32
	New York	39.0 (21.0)		43.0 (29.0)	0.93
	Pennsylvania	36.0 (29.0)		38.0 (29.5)	0.70
Overactive bladder	Top queried states:				
	South Dakota	13.5 (33.0)		7.5 (23.0)	0.22
	Arkansas	19.0 (30.0)		13.5 (22.5)	0.20
	Mississippi	10.0 (21.0)		18.5 (26.0)	0.05
	Delaware	3.0 (12.5)		6.0 (18.5)	0.20
	Nebraska	13.5 (25.0)		19.0 (30.0)	0.34
	Most populated states:				
	California	40.0 (27.0)		40.0 (20.5)	0.95
	Texas	35.0 (29.0)		35.0 (19.5)	0.56
	Florida	40.0 (26.0)		26.0 (26.5)	0.26
	New York	34.0 (29.0)		29.0 (22.5)	0.88
	Pennsylvania	35.0 (22.0)		35.0 (37.5)	0.90
Prostate cancer	Top queried states:				
	New York	64.0 (18.0)		62.0 (16.5)	0.25
	Kansas *	32.0 (22.0)		39.0 (16.0)	0.01
	Florida *	64.0 (16.0)		57.0 (21.0)	<0.01
	New Jersey	63.0 (20.0)		58.0 (22.5)	0.06
	Pennsylvania	54.0 (20.0)		54.0 (25.0)	0.66
	Most populated states:				
	California *	71.0 (12.0)		61.0 (14.0)	<0.01
	Texas *	60.0 (16.0)		53.0 (15.0)	<0.01
	Florida *	64.0 (16.0)		57.0 (21.0)	<0.01
	New York	64.0 (18.0)		62.0 (16.5)	0.25
	Pennsylvania	54.0 (20.0)		54.0 (25.0)	0.66
Urinary incontinence	Top queried states:				
	New Hampshire	19.0 (38.0)		16.0 (31.5)	0.11
	West Virginia	29.0 (54.0)		25.0 (49.5)	0.43
	Vermont	23.0 (28.0)		25.0 (31.0)	0.10
	Alabama	34.0 (29.0)		30.0 (27.5)	0.34
	Mississippi	18.0 (37.0)		28.0 (25.5)	0.71
	Most populated states:				
	California	64.0 (15.0)		63.0 (14.5)	0.97
	Texas	58.0 (21.0)		58.0 (20.5)	0.96
	Florida *	57.0 (16.0)		59.0 (16.5)	0.04
	New York	50.0 (19.0)		53.0 (25.0)	0.89
	Pennsylvania	46.0 (16.0)		42.0 (22.5)	0.41
Urinary tract infections	Top queried states:				
	Mississippi *	59.0 (20.0)		50.0 (22.0)	<0.01
	West Virginia	40.0 (18.0)		41.0 (19.5)	0.87
	Alabama *	76.0 (16.0)		70.0 (17.5)	0.04
	Arkansas	55.0 (17.0)		56.0 (14.5)	0.98
	Tennessee	66.0 (15.0)		66.0 (13.0)	0.46
	Most populated states:				
	California	81.0 (10.0)		81.0 (10.5)	0.82
	Texas	80.0 (9.0)		77.0 (9.0)	0.06
	Florida	81.0 (8.0)		79.0 (10.0)	0.05
	New York	78.0 (10.0)		75.0 (13.0)	0.07
	Pennsylvania *	76.0 (12.0)		71.0 (13.0)	<0.01

**Table 5:** Correlation of urologic condition searches to COVID-19 related searches during COVID in top queried and most populated states * Indicates p<0.05

Urologic condition	Location	R_S_	P
Benign prostatic hyperplasia	Top queried states:		
	Florida	-0.22	0.06
	Arizona	-0.02	0.85
	Connecticut	-0.06	0.61
	Mississippi	0.17	0.15
	Tennessee	-0.04	0.76
	Most populated states:		
	California	0.04	0.75
	Texas	-0.20	0.09
	Florida	-0.22	0.06
	New York	0.07	0.53
	Pennsylvania	-0.12	0.29
Bladder cancer	Top queried states:		
	Maine	-0.02	0.86
	New York	-0.10	0.40
	Vermont	0.16	0.17
	Connecticut	-0.19	0.11
	New Hampshire	-0.10	0.42
	Most populated states:		
	California *	-0.29	0.01
	Texas *	-0.25	0.04
	Florida	-0.11	0.33
	New York	-0.10	0.40
	Pennsylvania	0.08	0.50
Erectile dysfunction	Top queried states:		
	Louisiana	-0.06	0.63
	West Virginia	-0.18	0.12
	Mississippi	0.04	0.75
	Kentucky	-0.03	0.80
	South Carolina	0.17	0.15
	Most populated states:		
	California	0.10	0.94
	Texas	0.06	0.60
	Florida *	0.22	0.04
	New York	0.13	0.26
	Pennsylvania	0.12	0.30
Hematuria	Top queried states:		
	West Virginia	-0.18	0.12
	Mississippi	-0.15	0.19
	New Mexico	0.16	0.16
	North Dakota	-0.15	0.22
	Louisiana	-0.22	0.05
	Most populated states:		
	California	-0.07	0.56
	Texas	-0.10	0.40
	Florida	0.09	0.45
	New York	0.02	0.84
	Pennsylvania	-0.05	0.68
Interstitial cystitis	Top queried states:		
	South Dakota	-0.09	0.43
	Tennessee	0.04	0.84
	Kentucky	0.09	0.45
	Wyoming	-0.04	0.73
	Oklahoma	-0.13	0.27
	Most populated states:		
	California	0.07	0.55
	Texas	0.04	0.71
	Florida	-0.04	0.76
	New York	0.15	0.21
	Pennsylvania	0.13	0.25
Kidney cancer	Top queried states:		
	West Virginia	-0.03	0.80
	Vermont	0.14	0.22
	New York	0.13	0.28
	Wyoming	0.07	0.57
	Pennsylvania	0.06	0.60
	Most populated states:		
	California	0.07	0.96
	Texas	0.11	0.36
	Florida	-0.08	0.52
	New York	0.13	0.28
	Pennsylvania	0.06	0.60
Kidney stones	Top queried states:		
	West Virginia *	-0.26	0.03
	Kentucky	-0.22	0.06
	Tennessee	-0.06	0.62
	Alabama	-0.04	0.74
	Oklahoma	-0.12	0.33
	Most populated states:		
	California	-0.16	0.18
	Texas	0.10	0.38
	Florida	-0.01	0.94
	New York	0.09	0.45
	Pennsylvania	0.08	0.48
Low testosterone	Top queried states:		
	Wyoming	-0.05	0.87
	Alabama	-0.22	0.07
	Oklahoma	-0.03	0.80
	Mississippi	-0.04	0.77
	Tennessee	-0.11	0.37
	Most populated states:		
	California	-0.19	0.10
	Texas	-0.02	0.88
	Florida	-0.24	0.04
	New York	0.06	0.64
	Pennsylvania	-0.08	0.48
Overactive bladder	Top queried states:		
	South Dakota	0.02	0.89
	Arkansas	0.05	0.86
	Mississippi	0.07	0.55
	Delaware	-0.05	0.66
	Nebraska	0.11	0.34
	Most populated states:		
	California *	0.23	0.04
	Texas	-0.08	0.49
	Florida	0.06	0.58
	New York	-0.13	0.28
	Pennsylvania *	-0.24	0.04
Prostate cancer	Top queried states:		
	New York	-0.16	0.18
	Kansas	-0.19	0.11
	Florida *	-0.41	<0.01
	New Jersey	-0.17	0.15
	Pennsylvania *	-0.27	0.02
	Most populated states:		
	California *	-0.25	0.03
	Texas	-0.07	0.54
	Florida *	-0.41	<0.01
	New York	-0.16	0.18
	Pennsylvania *	-0.27	0.02
Urinary incontinence	Top queried states:		
	New Hampshire	-0.07	0.57
	West Virginia	0.09	0.46
	Vermont	0.07	0.55
	Alabama	-0.12	0.32
	Mississippi	0.01	0.98
	Most populated states:		
	California	0.07	0.95
	Texas	-0.15	0.21
	Florida	0.04	0.98
	New York	0.02	0.89
	Pennsylvania	-0.18	0.13
Urinary tract infections	Top queried states:		
	Mississippi	0.04	0.74
	West Virginia	-0.03	0.82
	Alabama	-0.04	0.75
	Arkansas	0.04	0.73
	Tennessee	0.04	0.75
	Most populated states:		
	California	-0.06	0.61
	Texas	0.08	0.49
	Florida	0.06	0.60
	New York	-0.06	0.64
	Pennsylvania	-0.14	0.22

## References

[1] De' R, Pandey N, Pal A (2020). Impact of digital surge during Covid-19 pandemic: a viewpoint on research and practice. Int J Inf Manage.

[2] Bento AI, Nguyen T, Wing C, Lozano-Rojas F, Ahn YY, Simon K (2020). Evidence from internet search data shows information-seeking responses to news of local COVID-19 cases. Proc Natl Acad Sci USA.

[3] Effenberger M, Kronbichler A, Shin JI, Mayer G, Tilg H, Perco P (2020). Association of the COVID-19 pandemic with internet search volumes: a Google Trendsᵀᴹ analysis. Int J Infect Dis.

[4] (2021). Google Trends: trends help. http://support.google.com/trends.

[5] (2021). Search engine market share united states of America. https://gs.statcounter.com/search-engine-market-share/all/united-states-of-america.

[6] Willard SD, Nguyen MM (2013). Internet search trends analysis tools can provide real-time data on kidney stone disease in the United States. Urology.

[7] Hesse BW, Nelson DE, Kreps GL, Croyle RT, Arora NK, Rimer BK, Viswanath K (2005). Trust and sources of health information: the impact of the Internet and its implications for health care providers: findings from the first Health Information National Trends Survey. Arch Intern Med.

[8] Pautler SE, Tan JK, Dugas GR, Pus N, Ferri M, Hardie WR, Chin JL (2001). Use of the internet for self-education by patients with prostate cancer. Urology.

[9] Lelie-van der Zande R, Koster ES, Teichert M, Bouvy ML (2021). Womens' self-management skills for prevention and treatment of recurring urinary tract infection. Int J Clin Pract.

[10] Dasgupta P (2020). Covid-19 and urology. BJU Int.

[11] Aitken RJ (2021). COVID-19 and human spermatozoa - potential risks for infertility and sexual transmission?. Andrology.

[12] Sansone A, Mollaioli D, Ciocca G, Limoncin E, Colonnello E, Vena W, Jannini EA (2021). Addressing male sexual and reproductive health in the wake of COVID-19 outbreak. J Endocrinol Invest.

[13] Black PC, Penson DF (2006). Prostate cancer on the internet--information or misinformation?. J Urol.

[14] Eysenbach G (2009). Infodemiology and infoveillance: framework for an emerging set of public health informatics methods to analyze search, communication and publication behavior on the Internet. J Med Internet Res.

[15] Dreher PC, Tong C, Ghiraldi E, Friedlander JI (2018). Use of Google Trends to track online behavior and interest in kidney stone surgery. Urology.

[16] Rezaee ME, Goddard B, Sverrisson EF, Seigne JD, Dagrosa LM (2019). 'Dr Google': trends in online interest in prostate cancer screening, diagnosis and treatment. BJU Int.

[17] Feinstein L, Matlaga B (2018). Urologic diseases in America. National Institute of Diabetes and Digestive and Kidney Diseases.

[18] Urology Care Foundation (2021). Urology Care Foundation: common conditions. https://www.urologyhealth.org/urology-a-z/all-conditions.

[19] (2021). WHO director-general's opening remarks at the media briefing on COVID-19. https://www.who.int/director-general/speeches/detail/who-director-general-s-opening-remarks-at-the-media-briefing-on-covid-19---11-march-2020.

[20] Moreland A, Herlihy C, Tynan MA (2020). Timing of state and territorial COVID-19 stay-at-home orders and changes in population movement - United States, March 1-May 31, 2020. MMWR Morb Mortal Wkly Rep.

[21] (2021). U.S. and world population clock (2021). Accessed: November 5. https://www.census.gov/popclock/.

[22] Ginsberg J, Mohebbi MH, Patel RS, Brammer L, Smolinski MS, Brilliant L (2009). Detecting influenza epidemics using search engine query data. Nature.

[23] Shamloul R, Ghanem H (2013). Erectile dysfunction. Lancet.

[24] Wang DE, Wadhera RK, Bhatt DL (2018). Association of rankings with cardiovascular outcomes at top-ranked hospitals vs nonranked hospitals in the United States. JAMA Cardiol.

[25] Nadim MK, Forni LG, Mehta RL (2020). COVID-19-associated acute kidney injury: consensus report of the 25th Acute Disease Quality Initiative (ADQI) Workgroup. Nat Rev Nephrol.

[26] Naspro R, Da Pozzo LF (2020). Urology in the time of corona. Nat Rev Urol.

[27] Teoh JY, Ong WL, Gonzalez-Padilla D (2020). A global survey on the impact of COVID-19 on urological services. Eur Urol.

[28] Sansone A, Jannini EA (2021). COVID-19 and erectile dysfunction: endothelial dysfunction and beyond. World J Mens Health.

[29] Tasnim S, Hossain MM, Mazumder H (2020). Impact of rumors and misinformation on COVID-19 in social media. J Prev Med Public Health.

